# The Aortic Pathologies: How Far We Understand It and Its Implications on Thoracic Aortic Surgery

**DOI:** 10.21470/1678-9741-2020-0089

**Published:** 2021

**Authors:** Amer Harky, Pawel Aleksander Sokal, Khubbaib Hasan, Andreas Papaleontiou

**Affiliations:** 1 Department of Cardiothoracic Surgery, Liverpool Heart and Chest Hospital, Liverpool, United Kingdom.; 2 School of Medicine, University of Liverpool, Liverpool, United Kingdom.; 3 Liverpool Centre for Cardiovascular Science, Liverpool Heart and Chest Hospital, Liverpool, United Kingdom

**Keywords:** Aorta Aneurysm, Aneurysm, Dissection, Vasculitis, Hypertension, Connective Tissue Diseases, Emergencies

## Abstract

Thoracic aortic diseases contribute to a major part of cardiac surgeries. The severity of pathologies varies significantly from emergency and life-threatening to conservatively managed conditions. Life-threatening conditions include type A aortic dissection and rupture. Aortic aneurysm is an example of a conservatively managed condition. Pathologies that affect the arterial wall can have a profound impact on the presentation of such cases. Several risk factors have been identified that increase the risk of emergency presentations such as connective tissue disease, hypertension, and vasculitis. The understanding of aortic pathologies is essential to improve management and clinical outcomes.

**Table:** 

Abbreviations, acronyms & symbols				
**AA**	**= Aortic aneurysm**		**MMPs**	**= Matrix metalloproteinases**
**AAA**	**= Abdominal aortic aneurysm**		**MRI**	**= Magnetic resonance imaging**
**AAS**	**= Acute aortic syndrome**		**NA**	**= No conclusive evidence**
**ACE**	**= Angiotensin-converting enzyme**		**NK**	**= Natural killer**
**AD**	**= Aortic dissection**		**NT-proBNP**	**= N-terminal pro B-type natriuretic peptide**
**ADCC**	**= Antibody-dependent cellular cytotoxicity**		**PAU**	**= Penetrating aortic ulcer**
**ATS**	**= Arterial tortuosity syndrome**		**PDFG**	**= Platelet-derived growth factor**
**BAV**	**= Bicuspid aortic valve**		**RNA**	**= Ribonucleic acid**
**CRP**	**= C-reactive protein**		**ROIs**	**= Regions of interest**
**CT**	**= Computer tomography**		**SGS**	**= Shprintzen-Goldberg syndrome**
**DEND**	**= Dendritic cell**		**TA**	**= Takayasu's arteritis**
**ECM**	**= Extracellular matrix**		**TAA**	**= Thoracic aortic aneurysm**
**EDS**	**= Ehlers-Danlos syndrome**		**TAAD**	**= Thoracic aortic aneurysm and dissection**
**FL**	**= False lumen**		**TEVAR**	**= Thoracic endovascular aortic repair**
**GC**	**= Giant cell**		**TGF-β**	**= Transforming growth factor beta**
**GCA**	**= Giant-cell arteritis**		**Th**	**= T helper**
**HLA**	**= Human leukocyte antigen**		**TL**	**= True lumen**
**HTAD**	**= Heritable thoracic aortic diseases**		**TLR**	**= Toll-like receptor**
**IFN-γ**	**= Interferon gamma**		**TNFα**	**= Tumour necrosis factor alpha**
**IL**	**= Interleukin**		**TOE**	**= Transoesophageal echocardiography**
**IMH**	**= Intramural haematoma**		**TS**	**= Turner syndrome**
**LDS**	**= Loeys-Dietz syndrome**		**VEGF**	**= Vascular endothelial growth factor**
**LPS**	**= Lipopolysaccharide**		**VSRR**	**= Valve-sparing root replacement**
**LVV**	**= Large vessel vasculitides**		**WSS**	**= Wall shear stress**
**MFS**	**= Marfan syndrome**			

## INTRODUCTION

Diseases affecting the thoracic aorta can be categorized into chronic and acute. The most common pathologies are thoracic aortic aneurysm and dissection (TAAD), acute aortic syndrome (AAS), connective tissue diseases, and vasculitis^[^^1^^]^. Despite the differences in pathophysiology of those diseases, hypertension and atherosclerosis are the main mechanisms behind their pathogenesis^[^^2^^]^. Cholesterol and fat accumulation within the arteries accelerate the breakdown of collagen and elastin, thereby compromising strength, structure, and elasticity of the aortic wall. Hypertension, cigarette smoking, family history, and age are the main contributors to atherosclerosis and progressive aortic disease.

An ongoing, progressive disease of the aorta is thoracic aortic aneurysm (TAA), which is defined as an increase of the aortic diameter by 1.5 times or more of its normal size. Inflammatory processes inside the vessel wall lead to loss of structural integrity and sequential dilatation, which in turn disrupts laminar flow and increases risk of thrombus formation^[^^3^^]^. In addition, the aneurysm can rupture and result in devastating consequences for the patient.

Another progressive connective tissue disease is Marfan syndrome (MFS). It significantly affects the aorta, which is exposed to high shear stress. Structural integrity of the aortic wall is exacerbated by impaired protein production, increasing the risk of aneurysm formation and dissection, even at a young age^[^^4^^]^.

Vasculitis is the inflammation of blood vessels and is an acute condition. The aetiology in many cases is unknown. Sometimes it can be attributed to recent infection, especially of a viral nature^[^^5^^,^^6^^]^. Thickening, narrowing, weakening, or scarring of the vessel wall is observed, which restricts blood flow. Takayasu's arteritis (TA) and giant-cell arteritis (GCA) are subtypes of vasculitis and affect larger arteries such as the aorta^[^^5^^]^.

Another acute condition affecting the aorta is aortic dissection (AD). A tear in the intima allows blood flow into the media resulting in the development of a true lumen (TL) and a false lumen (FL), separated by an intimo-medial flap^[^^7^^]^. Penetrating aortic ulcer (PAU), also an acute condition, is an outpouching of blood through the internal elastic lamina, typically arising from inflammatory erosion accompanying atherosclerotic plaque^[^^8^^]^.

## PHYSIOLOGY AND SHEAR STRESS OF THE THORACIC AORTA

The aorta, like all arteries, is composed of three main layers: the intima, media, and adventitia^[^^9^^]^. The intima provides a smooth surface for blood flow, the media allows expansion and contraction, whilst the adventitia provides support and structure to the artery. The detailed composition and function of each layer is summarised in Table 1.

**Table 1:** Composition and function of the layers of a blood vessel.

Layer	Composition	Function
Intima	It's the innermost layer and it is composed of a single layer of endothelial cells, a thin basal membrane, and a subendothelial layer of collagen fibrils	To provide a smooth surface for the blood to flow
Media	It's the middle layer and it is composed of elastic and collagen fibrils, smooth muscle, and elastic laminae separating the layer into transversely isotopic fibre-reinforced units	It allows expansion and contraction of the artery
Adventitia	It's the outermost layer, which is composed of thick bundles of collagen fibrils arranged in a helical structure and loose connective tissue	It provides support and structure

Blood flows in a pulsatile nature, stretching the aortic walls during systole and creating potential energy that will help maintain blood pressure during diastole^[^^10^^]^. The pressure exerted on the arterial wall is also known as the arterial blood pressure and is highest in the aorta. The wall shear stress (WSS) expresses force per unit area exerted by the wall on the fluid in direction of the local tangent plane. High WSS negatively affects the atherogenic process. Therefore, segments with low or oscillating WSS are at higher risk of atherosclerosis development, which results in a non-uniform distribution of atherosclerosis within arteries^[^^11^^]^. Dysfunctional (athero-prone) endothelial cells observed in areas of non-laminar flow have an activated, pro-inflammatory phenotype characterized by poor alignment, high turnover, and high oxidative stress^[^^12^^]^. This highlights the relationship between blood flow and atherosclerosis.

Alteration of the quantity and/or architecture of the connective fibres within the aortic wall directly impairs the elasticity and strength of the wall. Mechanical changes expose affected sections of the wall to non-laminar flow, and therefore can cause numerous pathologies. An example of this is AD. Typical locations of dissection highlight the link between pathology and mechanical weakness of the wall. Dissection typically occurs in the aortic root (type A) or at the end of the aortic arch (type B) where blood flow is less organized and disruptive to the integrity of the wall^[^^13^^]^.

## THE IMMUNOPATHOLOGY OF THORACIC AORTIC DISEASES

Atherosclerotic plaque formation is mediated by the immune system and significantly contributes to aortic disease. The first step of atherosclerosis is endothelial damage caused by sustained hypertension, smoking, drinking, and obesity. The endothelium is activated by expression of adhesion molecules; high levels of interferon alpha and beta are generated upon the activation of Toll-like receptor-9. T cells produce pro-inflammatory mediators such as interferon gamma (IFN-γ) and upregulate macrophages, which adhere to the endothelium and migrate into the intima^[^^14^^]^. Therefore, atherosclerotic lesions contain immune system cells alongside cholesterol deposits, which infiltrate from the blood.

The development of an aortic aneurysm (AA) depends on the interactions between the constituents of the aortic wall. Toll-like receptor-4 directly promotes inflammation by the upregulation of matrix metalloproteinases (MMPs) - MMP-2 and MMP-9 - in the aortic wall. T helper 1 cells secrete cytokines like interleukin (IL)-2 and IFN-γ, which have an anti-inflammatory function, while T helper 2 (Th2) cells mainly produce pro-inflammatory cytokines, including IL-4 and IL-5. A high ratio of Th2 cells in AA walls was observed, which contributes significantly to the disease pathology and secretion of extracellular matrix (ECM) degrading enzymes like neutrophil collagenase or protease^[^^15^^,^^16^^]^.

Inflammation of the aortic wall is the first step in AD. Inflammatory cells upregulate the expression of proteases and cell adhesion molecules. Additionally, they trigger a release of reactive oxygen species and induce apoptosis of smooth muscle cells, which leads to degradation of the wall^[^^17^^]^.

**Fig. 1:**
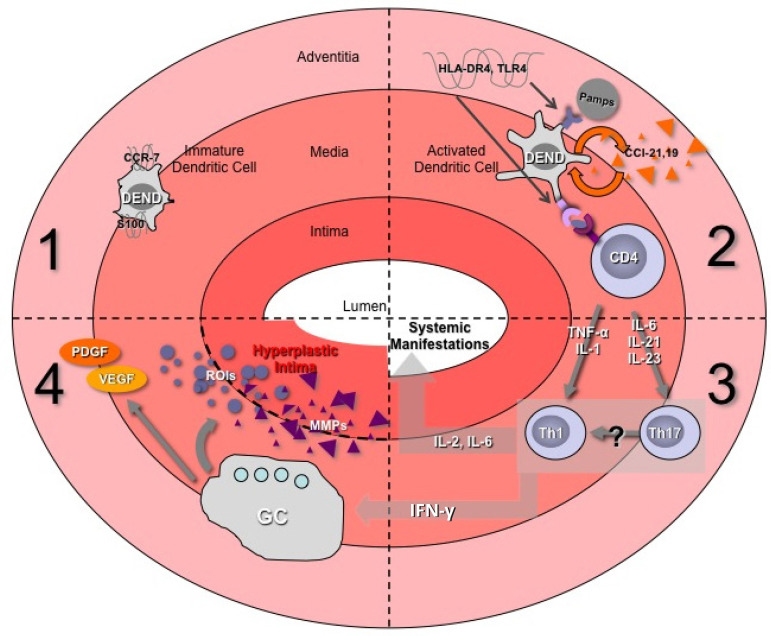
Immunopathology of the arterial wall in vasculitis. DEND=dendritic cell; GC=giant cell; HLA=human leukocyte antigen; IFN-γ=interferon gamma; IL=interleukin; MMPs=matrix metalloproteinases; PDGF=platelet-derived growth factor; ROIs=regions of interest; Th=T helper; TLR=Toll-like receptor; TNFα=tumour necrosis factor alpha; VEGF=vascular endothelial growth factor

Involvement of the immune system in vasculitides is noteworthy. GCA is characterized by a granulomatous inflammatory infiltrate involving the media, with a variable number of multinucleated giant cells. Tunica media injury induces the genesis of laminar medial necrosis^[^^18^^]^. The pathogenesis of GCA involves recruitment and stimulation of antigen specific T cells, IFN-γ upregulation, and activation of macrophages in the adventitial layer, which secrete IL-1, IL-6, and MMPs (Figure 1)^[^^5^^]^. In TA, the pathogenesis has not yet been elucidated (Figure 2). Despite that, it is known that the immune reaction targets the arterial wall, leading to progressive wall fibrosis, which ultimately results in luminal stenosis or aneurysm formation^[^^6^^]^. Lymphocyte subsets, macrophages, and pro-inflammatory cytokines (IL-6, IL-17, IFN-γ, and tumour necrosis factor alpha) are involved. The antigen(s) responsible for the autoimmune reaction remains unknown and its(their) identification would be of great benefit for improvement of immunomodulatory therapies^[^^6^^,^^19^^,^^20^^]^.

**Fig. 2:** Current understanding of the pathogenesis of Takayasu’s arteritis^[^^9^^]^. ADCC=antibody-dependent cellular cytotoxicity; IFN-γ=interferon gamma; IL=interleukin; LPS=lipopolysaccharide; MMPs=matrix metalloproteinases; NK=natural killer; Th=T helper; TLRs=Toll-like receptors; TNFα=tumour necrosis factor alpha.


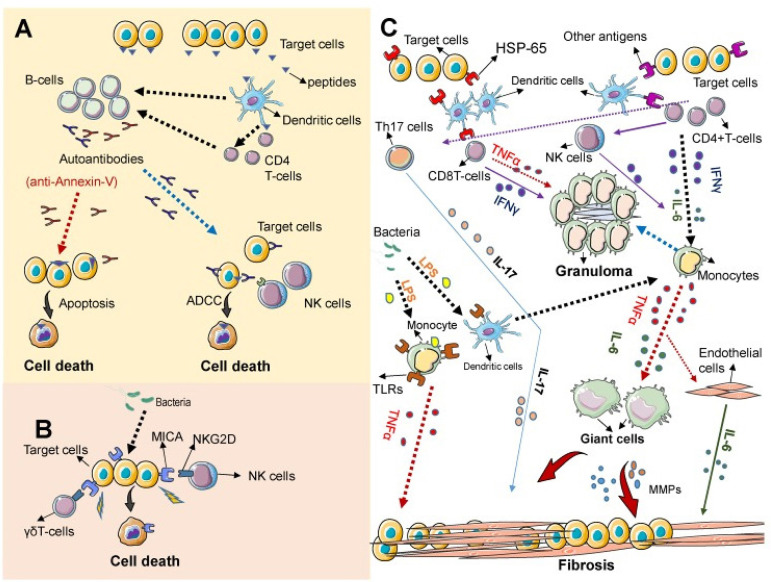


## ACUTE AORTIC SYNDROME

**Fig. 3:**
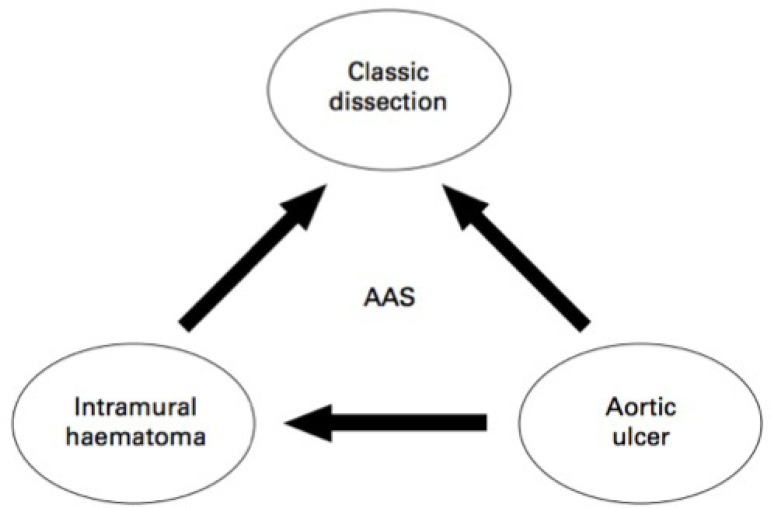
Illustrating diagram of acute aortic syndrome (AAS). Arrows indicate the possible progression of each of these aortic lesions^[^^24^^]^.

AAS encompasses PAU, AD, and intramural haematoma (IMH)^[^^8^^]^. Each of these conditions have their own unique pathogenesis, demographics, and outcomes, but they share similar clinical presentations: patients commonly present with “aortic chest pain” with similar radiological findings^[^^21^^]^. This is explained by the fact that all three conditions are a manifestation of an anatomical disturbance to the tunica media. The similar presentations make it hard to determine the exact pathophysiological mechanism behind the subset of AAS and there is a probable sequential link between these three conditions (Figure 3)^[^^21^^]^.

AD comprises the majority of cases of AAS and is characterised by severe, tearing chest pain radiating to the back^[^^7^^]^. It has the highest mortality of the three with 80% of patients expected to die by the start of the third week. The condition can be categorised as being acute or chronic; duration of symptoms lasting > 14 days is the margin, past which symptoms are chronic by definition. It can further be subcategorised according to the Stanford criteria as type A, which involves the ascending aorta, and type B, which does not. The Debakey type I dissection originates in the ascending aorta and propagates to or beyond the aortic arch, Debakey type II originates in and is confined to the ascending aorta, while Debakey type III originates in the descending aorta. AD occurs when a tear develops in the intimo-medial layer, thereby creating a channel for blood to enter the aortic wall: the FL. The FL most often traverses spirally due to the arrangement of layers in the media and it splits these layers apart from each other and the intima. Ultimately, this FL can compress the TL, leading to ischaemic symptoms or even rupture^[^^6^^]^. The most common risk factors for this disease are hypertension and smoking, however certain inherited conditions (*e.g*., MFS, Loeys-Diez syndrome [LDS], and Turner syndrome [TS]) can also lead to a diseased and weakened vessel, thereby making the aorta more susceptible to dissection^[^^7^^,^^9^^]^.

In a PAU, an atherosclerotic plaque ulcerates and sequentially penetrates the media, exposing it to high velocity blood flow, which may result in the formation of an IMH^[^^22^^]^. The haematoma may extend into the adventitia and form a pseudoaneurysm, which can rupture at any point^[^^22^^]^. PAU and AD share similar risk factors and presenting symptoms are identical; PAU can only definitively be diagnosed via computer tomography (CT), where a pouch-like protrusion of the aorta is seen without any FL or dissection flaps present. Transoesophageal echocardiography (TOE) will further help to visualise the jagged edged ulcer with the linked aortic atheroma and extensive calcification^[^^22^^]^.

IMH is a region of localised bleeding within the media but without initial intimal flap involvement. The pathogenesis of IMH remains unknown, however as with AD and PAU: atherosclerosis and hypertension play a significant part. Commonly, it is caused by some form of intrusion into the media leading to haemorrhage - normally by a PAU. This haemorrhage weakens the wall and the result is a confined blood filled region without intimal rupture or discontinuity^[^^23^^]^. If the wall is weakened enough, classic AD formation may occur and an estimated 10-30% of patients with AD have a pre-existing IMH^[^^23^^]^. The presentation is “aortic chest pain”, however patients with IMH are older than those with AD. As is the case with AD and PAU, it is life-threatening, so diagnosis should be rapid. CT (or, less routinely, TOE) can be used for definitive diagnosis if a wall thickness > 7mm is observed with an echolucent crescent shaped zone in the aortic wall, which is narrowing the aortic lumen^[^^23^^]^.

## VASCULITIS

Aortitis is an umbrella term used to describe inflammation of the aortic wall resulting from a plethora of histological pathologies^[^^21^^]^. GCA and TA are the two most common forms of arteritis arising from a non-infectious aetiology and both are classified as large vessel vasculitides (LVV)^[^^24^^]^. Aortitis is also associated with other non-infectious aetiologies such as: Cogan’s syndrome, sarcoidosis, Behcet’s disease, and systemic lupus erythematosus. Syphilis, tuberculosis, and salmonella are the most common infectious aetiologies of this disease^[^^24^^]^.

The exact pathogenesis of LVV is still not understood and although the two most common types of LVV share a similar inflammatory pathogenesis, their clinical presentations differ significantly. The main characteristics and differences of TA and GCA are summarised in Table 2. Both GCA and TA are inflammatory responses of large vessels that are granulomatous in nature, however they differ histologically. GCA involves minimal inflammation to the adventitia with the majority of inflammation occurring in the intima and media^[^^25^^]^. Calcium deposits accumulate in these two layers resulting in a “foreign body-like” inflammatory reaction in which granulomas that encapsulate the calcifications are formed^[^^26^^,^^27^^]^. TA, however, mostly involves the adventitia where two distinct histopathological stages occur. The first stage (the acute inflammatory phase) involves the vasa vasorum of the adventitia being surrounded and infiltrated by plasma cells and lymphocytes, and later multinucleated giant cells^[^^24^^]^. The second stage (the chronic fibrotic stage) occurs as a result of chronic inflammation, which ultimately leads to fibrotic plaque formation and thickening of the vessel wall^[^^24^^]^. Consequentially, the aortic lumen narrows in a “skip” pattern. Therefore, the thickness of the aortic wall is characteristically greater in TA patients than in GCA patients.

**Table 2:** Summary table highlighting the differences between Takayasu's arteritis and giant-cell arteritis.

	Takayasu's arteritis	Giant-cell arteritis
Peak age of onset, years	15-30	60-80
Female:male ratio	9:1^[^^86^^]^	03:01
Racial predisposition	Eastern Asia (Japan)^[^^87^^]^	Caucasian (northern Europe)
Common presentation	Symptoms of aortic branch occlusion	Headache
		Visual impairment
Aorta affected, %	100%	40%
Common histological appearance	Excessive adventitial fibrosis Well-circumscribed intimal fibrocellular hyperplasia Increased vessel wall thickness	Prominent inflammation of inner media and intima
		Fragmentation of internal elastic lamina
		Focal aortic wall inflammation with "skip lesions"
Common genetic association	HLA-B52	HLA-DR4
	Non-HLA (ILI2B region)	
Immunopathological features	Activation of adventitial dendritic cells	
	Recruitment of activation of CD4+ T lymphocytes	
	Macrophage activation with giant cell formation and release of pro-inflammatory cytokines	
	Neovascularisation and intimal hyperplasia	
Aetiological trigger	Unknown: maybe infective agent or autoantigen	

HLA=human leukocyte antigen

In both diseases, CD4+ T cells proliferate and infiltrate the media simultaneously, releasing a cascade of pro-inflammatory cytokines, which trigger further differentiation of the CD4+ T cells^[^^28^^,^^29^^]^. Ultimately, this inflammation leads to remodelling of the vessel wall resulting in stenosis and aneurysm formation. MMPs are mediators of vascular remodelling, which degrade components of ECM^[^^30^^]^. In one study, a significantly higher messenger ribonucleic acid expression of all MMPs, including MMPs- 1, 2, and 3, was observed in patients with TA compared to healthy subjects. Therefore, ECM remodelling was advanced, with disruption of elastic fibres, making these patients more susceptible to atherosclerosis and aneurysms^[^^31^^]^. Furthermore, the production of angiogenic factors in the affected vascular areas results in neovascularization: providing novel routes of entry for leukocytes^[^^32^^]^.

The inflammation, remodelling, and consequent vascular stenosis in both diseases lead to classical clinical presentations. In TA, the aorta, aortic arch, or other large vessels are implicated, leading to TA, also being referred to as aortic arch syndrome and pulseless disease. The majority of patients present with occlusion of the suprarenal/renal arteries and aorta resulting in hypertension, bruits, extremity claudication, and pulse deficits^[^^24^^]^. A vascular examination with blood pressure measurement is essential in the diagnosis of TA to detect this characteristic extremity involvement^[^^24^^]^. Angiography will reveal the abdominal aorta to be the most common site of involvement in TA, followed by the descending aorta and arch. Extensive stenosis is visible, with AAs being common and reported in 45% of patients^[^^24^^]^. The diagnosis of GCA is made by specific criteria established particularly for its diagnosis by the American College of Rheumatology (Table 3), which has 91% specificity and 94% sensitivity^[^^33^^]^. Radiological findings include long segment, tapering lesions in the subclavian, and axillary arteries with thickening of the vessel wall^[^^34^^]^.

**Table 3:** Criteria for diagnosis of giant-cell arteritis.

Criterion	Definition
Age of patient at disease onset in years	Development of symptoms (headache, scalp tenderness, polymyalgia rheumatica, jaw pain, and vision changes) or findings related to giant-cell arteritis at age < 40 years
Claudication of extremities	Development and worsening of fatigue and discomfort in muscles of one or more extremity while in use, especially the upper extremities
Decreased brachial artery pulse	Decreased pulsation of both brachial arteries
Blood pressure difference -10 mmHg	Difference of > 10 mmHg in systolic blood pressure between arms
Bruit over subclavian arteries or aorta	Bruits audible on auscultation over one or both subclavian arteries or abdominal aorta
Arteriogram abnormality	Arteriographic narrowing or occlusion of the entire aorta, its primary branches, or large arteries in the proximal upper or lower extremities, not due to arteriosclerosis, fibromuscular dysplasia, or similar causes. Changes are usually focal or segmental.

Giant-cell arteritis is classified if at least three of the six criteria are present.

GCA and TA are both systemic diseases with multifocal implications and both have atypical presentations. GCA has been found to atypically present as uveitis (particularly in elderly patients), due to vasculitis of the posterior ciliary arteries, or as stroke, headache and encephalopathy due to cerebral vasculitis (rare and < 2%)^[^^35^^]^. TA has been found to atypically present as a hypertensive emergency and as refractory abdominal pain^[^^36^^,^^37^^]^.

## GENETICS AND ITS ROLE IN AORTIC PATHOLOGIES

**Fig. 4:**
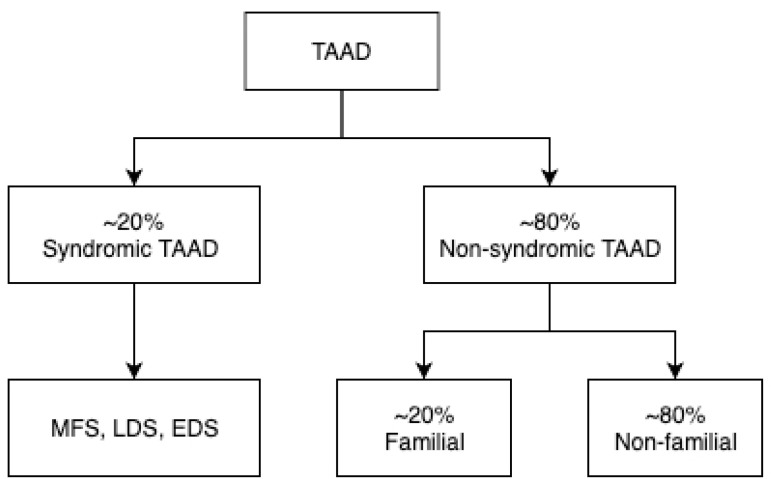
Classification of thoracic aortic aneurysm and dissection (TAAD) where percentage values refer to approximate prevalence. EDS=Ehlers-Danlos syndrome; LDS=Loeys-Dietz syndrome; MFS=Marfan syndrome^[^^39^^]^.

Genetics, unequivocally, plays an inextricable role in the development of thoracic aortic pathologies including acute AD, PAU, IMH, and TAAD. Classification of TAAD (Figure 4) depends on extra-aortic pathology presence (syndromic TAAD) or absence (non-syndromic TAAD). Syndromic TAAD examples are MFS, LDS, and Ehlers-Danlos syndrome (EDS). These are all types of connective tissue disorders leading to aortopathies displaying a common final aberration - overstimulation of transforming growth factor beta (TGF-β) activity in the ascending aorta -, resulting in them being referred to as the TGF-β vasculopathies^[^^38^^]^. However syndromic TAADs are responsible only for 20% of all TAAD cases. Consequently, non-syndromic TAAD comprises the majority of TAAD cases and can be further split into familial non-syndromic TAAD (one or more family members with the disease - approximately 20% prevalence) and non-familial TAAD (no family members with the disease). Interestingly, in both syndromic and non-syndromic TAAD cases, very often only one gene seems to be responsible for the pathophysiological mechanism of the disease. This creates potential for pre-emptive detection and treatment, in contrast to diseases which have multi-genetic and complex pathophysiological geneses, such as atherosclerosis^[^^39^^]^.

MFS is caused by a mutation in the FBN1 gene, which impairs function of a protein called fibrillin-1. The dysfunctional fibrillin-1 contributes to upregulated signalling activity of the TGF-β1 protein, which in turn promotes production of pro-inflammatory transcription factors. As a consequence, upregulation of MMPs (*e.g*., MMP-2 and 9) and cytokines leads to progressive vascular wall remodelling, deterioration of aortic mechanical properties, and contractility of smooth muscle cells. The tunica media and adventitia become infiltrated with T lymphocytes and macrophages. Additional histological findings in the tunica media are cystic medial necrosis with loss of nuclei, accumulation of amorphous matrix components, fibrotic collagen production, focal interlamellar elastic fibre degradation, and decreased expression of α-smooth muscle actin, smooth muscle protein 22α, and smoothelin^[^^20^^]^. Additionally, TGF-β promotes accumulation of reactive oxygen species or nitrogen intermediates, which contribute to further fibre degradation. Recent studies identified a number of newly discovered molecular mechanisms, which interact with the TGF-β pathway including miR-29b, SMAD, activating protein-1, nicotinamide adenine dinucleotide phosphate oxidase-4, and the mTOR pathway^[^^40^^]^. The ultimate result of these changes is type A AD, with 70% lifetime prevalence^[^^41^^]^. In terms of clinical management, currently AA in MFS should be surgically repaired when 4.5-5.0 cm in diameter or earlier if the aneurysm is growing at a high rate (MFS aneurysms grow at 0.26 cm/year) or in patients planning pregnancy^[^^39^^]^.

LDS is caused by a mutation in one or more of the following genes - TGFBR1, TGFBR2, SMAD3, and TGFB2 -, and results in generalised arterial tortuosity, formation of an ascending AA, and dissection at an early age due to TGF-β pathway upregulation. Histologic findings of LDS aortas show diffuse medial degeneration with loss or fragmentation of intralamellar elastic fibres resulting in generalised widening of intralamellar spaces in tunica media^[^^42^^]^. These changes are observed without any signs of inflammation, which suggests that the main pathophysiological mechanism is a severely impaired production of elastin: this contrasts with MFS in which the destruction of elastic fibres is secondary. Samples from LDS patients are consistent with the mechanism of TGF-β pathway overstimulation^[^^43^^]^. Cardiovascular implications are more severe than in MFS, with dissections and ruptures occurring at a younger age and at a smaller diameter^[^^44^^]^. Overall, 32% of patients with this condition go on to develop thoracic AD. It can be differentiated from MFS as it has some exclusive clinical features (clubfoot, craniosynostosis, cervical spine instability) and the aforementioned manifestation of vascular complications at a younger age^[^^44^^]^. For this reason, current guidelines recommend surgical repair at 4.0 to 4.2 cm aortic diameter^[^^39^^]^.

Upregulated TGF-β signaling in Shprintzen-Goldberg syndrome (SGS) is a similar finding to that in MFS and LDS. In SGS, a mutation of the SKI proto-oncogene protein is the culprit. However, only a single report of aortic root dissection has been reported in literature and the histology of the aorta has not been described^[^^45^^]^.

Vascular EDS starts as arterial fragility, which leads to aneurysm formation, arteriovenous fistulae, vascular dissection, and rupture. Mutation of the COL3A1 gene impairs the regulation of TGF-β bioavailability and results in ECM disarray due to impaired ECM remodelling and collagen biosynthesis, generalised fibrillin disarray, disorganisation of proteoglycans, altered endoplasmic reticulum homeostasis, as well as poor protein quality control. These all significantly decrease the stability of the tunica media and adventitia of blood vessels^[^^46^^]^. Histologic examination shows minimal medial degeneration of the aorta and partial disruption of elastic laminae with fibrous tissue deposits. Irregular diameters of collagen fibres and fibrinogranular substance in the ECM can be present^[^^42^^]^. Vascular rupture and dissection can occur in 80% of patients before the age of 40 and 50% of these cases occur in the abdomen and thorax^[^^47^^]^. In terms of clinical management, prophylactic intervention is advised at an aortic diameter between 4.5 to 5.0 cm^[^^39^^]^.

EDS clinically overlaps with arterial tortuosity syndrome (ATS), however ATS involves no structural defect to collagen. In ATS, the mutated GLUT10 glucose transporter gene contributes to upregulation of the TGF-β pathway in the vessel^[^^48^^]^. ATS patients are likely to develop aneurysms (19-31% of patients) and arterial dissection (which is suspected to be a result of higher shear stress). Histological examination of the vessel wall reveals fragmentation of the inner elastic membrane, degradation of the elastic fibres of the tunica media, and loss of the external elastic membrane. The intima of the vessels is also fibrotic and significantly thicker than normal^[^^42^^]^.

TS is characterised by a complete or partial absence of one X chromosome (45, X karyotype) and is the most common cause of AD in young women (the risk is increased by more than 100)^[^^49^^]^. TS can also result in congenital heart defects including aortic coarctation (12%) and bicuspid aortic valve (30%)^[^^49^^]^. The increased risk of AD has commonly been attributed to the increased incidence of aortic coarctation in TS, although there are ongoing studies to establish whether there is a Marfan-like aortopathy that increases risk of macrovascular defects rather than confinement of causation to aortic coarctation^[^^49^^]^. Approximately 1% of patients with aortic coarctation go on to develop AD, however estimates of the incidence of AD in patients with TS vary considerably^[^^50^^]^. Operative management of the aortic root and ascending aorta is recommended for women who are ≥ 15 years of age, have an ascending aortic size index ≥ 2.5 cm/m^2^, and have associated risk factors for AD, including bicuspid aortic valve and hypertension. Additionally, surgical consultation is advised if an increase in aortic diameter of > 0.5 cm/year is recorded^[^^51^^]^.

Despite separate classification, there is considerable overlap between syndromic and non-syndromic TAADs as the same mutation can cause syndromic features in one patient whilst not completing the full syndrome in others^[^^52^^]^. In the non-syndromic group, 30% of inherited TAAs could be attributed to a causative mutation in one of the heritable thoracic aortic diseases genes (Table 4)^[^^53^^]^.

**Table 4:** The HTAD genes.

Gene (locus)	Proportion of families with HTAD and with a pathogenic variant in this gene	Syndrome	Other cardiovascular findings observed
ACTA2	12-21%	Multisystem smooth muscle dysfunction syndrome	
BGN	Rare	Meester-Loeys syndrome (OMIM 300989)	
COL3A1	Rare	Ehlers-Danlos syndrome type IV	
FBN1	3%	Marfan syndrome	
FOXE3	1.4%		
LOX	1.5%		Bicuspid aortic valve, abdominal aortic aneurysm, hepatic artery aneurysm
MAT2A	1%		Bicuspid aortic valve
MFAP5	0.25%		Atrial fibrillation, mitral valve prolapse, and arterial tortuosity in some patients
MYH11	1%		Patent ductus arteriosus
MYLK	1%		
PRKG1	1%		Coronary artery aneurysm/dissection and arterial tortuosity in some patients
TGFB2	1%	Loeys-Dietz syndrome	Abdominal aortic aneurysms and/or intracranial and other arterial aneurysms and/or dissections
TGFB3	2 simplex cases, one 3-generation family	Rienhoff syndrome or Loeys-Dietz syndrome type 5	
TGFBR1	3%	Loeys-Dietz syndrome	Abdominal aortic aneurysms and/or intracranial and other arterial aneurysms and/or dissections
TGFBR2	5%	Loeys-Dietz syndrome	
SMAD3	2%	Aneurysms osteoarthritis syndrome; Loeys-Dietz syndrome	
(AAT1 or FAA1)	Unknown		
AAT2 or TAAD1	Unknown		

HTAD=heritable thoracic aortic diseases

Familial non-syndromic TAAD follows an autosomal dominant pattern of inheritance with reduced penetrance and variable expressivity^[^^52^^]^. Genes like ACTA2, MYLK, MYH11, and PRKG1 were established to play a role in this condition and their mutations to cause AD at a diameter < 5.0 cm. Additionally, positive family history significantly increases risk of AD and TAAD severity, which manifests as a younger age of first presentation (58.2 *vs.* 65.7 years) and faster growth rate (0.21 *vs.* 0.16 cm/year)^[^^39^^]^. Histological findings from the aortas of patients with MYH11 and ACTA2 mutations show focal medial degeneration with disarrayed vascular smooth muscle cells, degeneration of elastic fibres, and increased penetrance of vasa vasorum into the tunica media^[^^42^^]^. As far as clinical management is concerned, Ostberg et al.^[^^39^^]^ proposed a pathway for relatives of a non-syndromic TAAD patient. It recommends surgical repair under these conditions: if possible genetic carrier of specific named mutations or positive family history of dissection or other cardiovascular disease at aortic diameter > 4.2 cm or growth > 3 mm/year; if negative genetic carrier and aortic diameter > 5.0 cm or growth > 5 mm/year.

Non-familial and non-syndromic TAAD is referred to as sporadic TAAD. Until recently, sporadic TAAD was regarded as degenerative only, however current research aims to identify a genomic component involved in sporadic TAAD development - FBN1, TGFBR, TGFBR2, COL3A1, SMAD, ACTA2, and TGFB2 genes are being investigated^[^^39^^]^.

In all cases, the risk of AAS can be increased by the excessive production of MMPs: MMPP-1, MMP-9, and MMP-12^[^^54^^]^. These can all lead to accelerated degradation of elastin and collagen fibres, which results in vascular wall weakness. It follows that mutations, which lead to the excessive production of these MMPs, increase the risk of MFS and its associated vascular complications and AAs, directly. In addition, non-syndromic mutations to genes such as MYH11, ACTA2, MYLK, and PRKG1 can directly lead to abnormalities in the contractile mechanism of smooth muscle as smooth muscle cells stop producing contractile proteins^[^^54^^]^. Consequently, in the presence of these mutations, there is an increased risk of thoracic AD either as an isolated manifestation or as a symptom of a syndrome.

Finally, there are racial differences for risk of AAS and TAAs, most of which relate to racial predispositions for the risk factors for these two conditions, such as hypertension, of which 77% of patients have a history of^[^^8^^]^. African patients are more likely to be younger and be affected by type B acute AD. Hypertension and cocaine abuse have been recognised as the predominant causes of acute AD in the black population^[^^8^^]^. Table 5 is a summary of genetic and non-genetic causes of thoracic aortic pathologies.

**Table 5:** Genetic and non-genetic causes of thoracic aortic pathologies.

	Causative genetic disorders	Other
Thoracic aortic aneurysm	Ehlers-Danlos syndrome	Non-syndromic mutations to genes such as MYH11 and ACTA2
	Marfan syndrome	Mutations leading to excessive production of MMPs
	Loeys-Dietz syndrome	Mutation in one of the heritable thoracic aortic diseases (HTAD) genes
	Turner's syndrome	Racial differences relating to hypertension
Acute aortic syndrome	Bicuspid aortic valve	Non-syndromic mutations to genes such as MYH11 and ACTA2
		Mutations leading to excessive production of MMPs
		Racial differences relating to hypertension

HTAD=heritable thoracic aortic diseases; MMPs=matrix metalloproteinases

## BIOMARKERS OF THORACIC AORTA

There is great interest in identifying a biomarker that will enable early detection of thoracic aortic pathologies like acute AD and TAA. According to Balmforth et al.^[^^55^^]^, the ideal biomarker would be easily detectable in peripheral blood before dissection or rupture of the aneurysm ensues and its levels would be proportional to the size of aortic dilatation. To this date, there is no biomarker which can be used for screening of asymptomatic patients. Existing candidates for biomarkers can be divided into four categories: generalised laboratory tests, focused TAA biomarkers, genetic markers, and novel biomarkers.

Generalised laboratory tests like D-Dimer, C-reactive protein, IL-6, leucocytes, plasma homocysteine, or lipoprotein (a) were found to correlate with presence of an abdominal aortic aneurysm (AAA) and/or TAA. However, they are also known to be linked with a myriad of other medical conditions, thus their specificity is very low and can only be used to exclude an aortopathy in suspected, symptomatic patients^[^^52^^,^^56^^,^^57^^]^.

Focused biomarkers of TAA are promising due to their high specificity, guaranteed by their direct involvement in the pathological process of TAA development. Some examples of such biomarkers include collagen V and XI, which showed to be upregulated in TAA, while collagen I and III are at lower level. Some forms of relevant proteins have been linked to be useful in TAA such as four and a half LIM domains protein 1, or FHL1, which was found to be a useful whole blood biomarker for TAA^[^^55^^]^. Other focused biomarkers include the renin-angiotensin system involvement in the pathogenesis of TAA, which was established, but its clinical significance is yet to be determined^[^^58^^]^. The impairment of ECM plays an important role in the pathogenesis of AA, thus a detectable biomarker corresponding to the difference of ECM composition between healthy and aorthopathy subjects would be of high clinical significance. Entire molecules or fragments of proteins like collagen, elastin, or fibrillin are potential biomarkers that need further investigation and clinical trials^[^^55^^]^. However, degradation of the aforementioned proteins is regulated by MMPs and tissue inhibitors of metalloproteinases. A difference in expression as well as plasma levels of these regulatory proteins between AA patients and healthy controls has been found: Li et al. recorded increased MMP-9 levels in TAA and AAA patients compared to healthy controls^[^^59^^]^. MMP-9 was described as having a strong diagnostic value for TAA with 70% sensitivity and 91% specificity. This method needs further prospective studies incorporating a larger sample size^[^^58^^]^.

Genetic biomarker candidates utilise a potential difference in the transcriptome of cells found in peripheral blood^[^^55^^]^. Genes like CLU, DES, MYH10, FBLN5, or TGF-β pathway were demonstrated to be involved in TAA formation and need to be explored for clinical relevance. A 41-gene signature in peripheral blood cells has been shown to help identify a TAA from a group of TAA patients and healthy controls with 80% accuracy^[^^60^^-^^62^^]^. Micro-ribonucleic acids, like miR-574-5p, miR-122-3p, and miR-483-3p, and molecules, such as Krüppel-like factor 4, can be potentially used as biomarkers after clinical testing^[^^39^^]^.

Novel biomarkers like osteopontin, endothelins, platelet-derived growth factor B, or a “leucocyte mean telomere length measurement” need further investigation to validate their involvement and/or correlation with aortopathies and clinical trials to determine their relevance^[^^57^^,^^63^^,^^64^^]^. Table 6 is a summary of potential TAA biomarkers based on van Bogerijen GHW et al.^[^^57^^]^.

**Table 6:** Potential TAA biomarkers.

Biomarker	Use for diagnosis of aneurysm	Use for prediction of growth or rupture	Aortic segment involved	Comments
D-Dimer	No	No	AAA, TAA	Can be used to exclude acute aortic event in symptomatic patients
CRP, IL-6, and leucocytes	Possible	Possible	AAA, TAA	Non-specific
Homocysteine	NA	NA	TAA	Correlates with degree of atherosclerosis
Lipoprotein (a)	NA	NA	TAA	Needs further investigation
Collagen markers	Possible	NA	TAA	Tested clinically in a small study
Elastin markers	Potential	Potential	AAA	Needs to be tested in TAA
Fibrillin	Potential	Potential	TAA	Need further investigation
MMPs	Possible	NA	AAA, TAA	Strong diagnostic value shown in a small study, possibly useful for evaluation of success of treatment
ACE	NA	NA	AAA, TAA	Needs further investigation and clinical trials
41-gene expression signature	Possible	NA	TAA	Promising, needs further clinical trials
TGF-β	Potential	NA	AAA, TAA	Needs clinical trials
CLU, DES, MYH10, and FBLN5 genes	Possible	NA	TAA	Needs clinical trials
Deregulated micro RNA and messenger RNA^[^^88^^]^	NA	NA	TAA	Needs clinical trials
Mean telomere length of leucocytes	NA	NA	TAA	Sporadic TAA detection^[^^57^^]^
Endothelin	Possible	Possible	AAA	Correlation with size of aneurysm
Fibulins	NA	NA	TAA	Potential detection of TAA at an early stage^[^^57^^]^
Filamins	NA	NA	TAA	Correlation with connective tissue disorders and BAV^[^^57^^]^
Osteopontin	NA	NA	TAA	Needs further investigation and clinical trials
Platelet-derived growth factor B	Possible	NA	TAA	Needs further investigation and clinical trials
Haemostatic markers (plasminogen, fibrinogen)	NA	NA	AAA	Distinguish acutely symptomatic non-ruptured AAAs from ruptured AAAs^[^^57^^]^
Thrombospondin-2^[^^57^^]^	NA	NA	TAA	
Interferons	NA	NA	TAA	Potentially useful in early detection of TAA^[^^57^^]^
SMAD	NA	NA	TAA	Presence in familial TAAs and in aneurysm osteoarthritis syndrome
NT-proBNP	NA	NA	TAA	May detect TAAs^[^^57^^]^

AAA=abdominal aortic aneurysm; ACE=angiotensin-converting enzyme; BAV=bicuspid aortic valve; CRP=C-reactive protein; IL=interleukin; MMPs=matrix metalloproteinases; NA=no conclusive evidence; NT-proBNP=N-terminal pro B-type natriuretic peptide; RNA=ribonucleic acid; TAA=thoracic aortic aneurysm; TGF-β=transforming growth factor beta

## MANAGEMENT OF THORACIC ARTERIAL DISEASE IN CURRENT ERA

There are a multitude of arterial diseases, the common ones include TAA, AD, IMH, and PAU. One mutual management aim is to reduce the manifestation of symptoms. This can be achieved by tackling the causative agent (possibly reducing exposure to the risk factor) followed by therapeutic and then surgical intervention with close monitoring.

Initial TAA management addresses risk factors like smoking, hypertension (< 140/90 mmHg target) and dyslipidaemia (lipid lowering agents reduce necessary surgical interventions by 44%)^[^^65^^-^^67^^]^. Surgical intervention is required when symptoms suggesting TAA expansion are present and when the risk of rupture or dissection exceeds the risk of the intervention^[^^68^^]^. The size thresholds for the intervention are governed by the pathogenesis and aetiology of the aneurysm, with a lower threshold for those with genetic aortopathies (Table 7)^[^^68^^]^.

**Table 7:** Class I and Class IIa recommendations on maximal aortic diameter cutoff for treatment of aortic aneurysm in asymptomatic patients^[^^67^^,^^89^^]^.

Ascending aortic or aortic sinus aneurysm	
≥ 55 mm	▸Patients with no aortopathy
	▸BAV without risk factors*
≥ 50 mm	▸BAV with risk factor*
	▸Marfan syndrome with no other risk factor†
≥ 45 mm	▸Marfan syndrome with risk factors†
	▸Patients with BAV undergoing surgical aortic valve repair
≥ 44-46 mm	▸LDS measured with TOE
≥ 42 mm	▸LDS measured with CT or MRI
**Aortic arch aneurysm**	
≥ 55 mm	Isolated arch aneurysm (may be planned earlier if adjacent ascending or descending thoracic aortic aneurysm repair is planned)
**Descending thoracic aortic aneurysm**	
≥ 55 mm	If anatomy is suitable for thoracic endovascular stent repair
≥ 60 mm	Surgical repair when anatomy is not suitable for stent repair

•*Coarctation of the aorta, systemic hypertension, family history of dissection or increase in aortic diameter > 3-5 mm/year (measured at the same level). •†Family history of aortic dissection, aortic sizea increase > 3-5 mm/year (measured at the same level), severe aortic or mitral regurgitation, or desire for pregnancy. BAV=bicuspid aortic valve; CT=computer tomography; LDS=Loeys-Dietz syndrome; MRI=magnetic resonance imaging; TOE=transoesophageal echocardiography

Surgical management of ascending AA is guided by the aneurysm location and underlying pathology. Segmental graft replacement is recommended for aneurysms distal to the sinotubular junction. In patients with aortic root dilatation and aortic valve regurgitation, aortic valve repair and root-sparing procedures might be used. If the aneurysm involves the aortic root, in patients with MFS, total replacement of the ascending aorta is required with either valve replacement (Bentall procedure) or valve-sparing root replacement (VSRR). Bentall and VSRR patients were found to have similar late survival outcomes and freedom from root reoperation, however VSRR procedures result in significantly fewer thromboembolic and hemorrhagic events^[^^69^^]^. Interestingly, minimally invasive endovascular ascending TAA repair has been performed for selected high-risk patients with satisfactory outcomes^[^^70^^]^.

TAA with dilation of the proximal aortic arch should be managed by partial arch replacement and ascending aorta repair via open surgery. Aortic arch aneurysms should be replaced entirely if the entire arch segment is affected. In the case of distal arch aneurysm, which also involves the proximal descending thoracic aorta, the elephant procedure is recommended. Cardiopulmonary bypass as well as some degree of hypothermia are required in aortic arch replacement. Hypothermia alone, direct antegrade perfusion through brachiocephalic arteries, or retrograde perfusion through the superior vena cava are used to protect the brain tissue during the cardiac arrest period. Despite all the protective measures, such procedures still entail higher operative mortality and stroke rates than interventions on isolated aneurysms of the ascending or descending thoracic aorta^[^^67^^]^. Additionally, aortic arch repair can be achieved using the thoracic endovascular aortic repair (TEVAR), where a stent is deployed inside the diseased vessel^[^^71^^]^. When compared to open surgery, it showed similar operative outcomes and long-term survival, however TEVAR was found to have inferiority of freedom from aortic reintervention^[^^72^^]^.

Descending thoracic aorta and thoracoabdominal AA can be repaired via TEVAR or open surgery^[^^71^^]^. Both procedures can be performed with similar operative outcomes, acceptable morbidity and mortality, and with low rates of postoperative complications^[^^73^^,^^74^^]^. Arnaoutakis et al.^[^^71^^]^ reports TEVAR to have decreased 30-day mortality and fewer major adverse events compared with open surgery.

Both AD and IMH are classed as type A if the ascending aorta is involved or type B if it is not involved, using the Stanford classification system^[^^75^^]^. In both types, medical intervention is essential to address pain and previous hypertension (systolic target is 100-120 mmHg and heart rate around 60 bpm)^[^^75^^]^. Medical management is the gold standard treatment for uncomplicated type B dissection, alleviating haemodynamic stress on the diseased and weakened vessel wall^[^^76^^]^. Uncomplicated type B dissections have a 30-day morality of < 10%, which is similar to surgical outcomes, making surgical intervention less beneficial^[^^76^^]^. Recently, TEVAR was compared to medical treatment of uncomplicated type B AD in a randomised control trial, which demonstrated that patients with medical management had less FL thrombosis (97% for TEVAR *vs*. 43% for medical treatment, *P*<.001), which is an important positive prognostic factor^[^^77^^,^^78^^]^. Additionally, TEVAR patients were found to have more favourable aortic remodelling (mean FL diameter, 18.5 mm *vs*. 25.1 mm, respectively; *P*<.001; maximum TL diameter, 32.2 mm *vs*. 25.5 mm, respectively; *P*<.001 for TEVAR and medical treatment)^[^^77^^]^. Therefore, current recommendation of Mussa et al. is TEVAR or medical therapy for uncomplicated type B aortic dissection^[^^77^^]^.

Complicated type B dissections require urgent endovascular repair or surgery^[^^75^^,^^79^^]^. Moulakakis et al.^[^^79^^]^ report superior 30-day/in-hospital survival for acute complicated type B AD managed by TEVAR compared to surgical aortic reconstruction (7.3% *vs*. 19.0%, respectively). Mussa et al. found 30-day/in-hospital mortality for type B acute AD of 0% to 27% (median, 7%) for medical treatment, 13% to 17% (median, 16%) for open surgical procedure, and 0% to 18% (median, 6%) for TEVAR. It is worth remembering that endovascular repair is not feasible in all patients and there are concerns with regards to the durability of this procedure^[^^77^^]^.

Surgical intervention is the gold standard for type A AD as mortality is 50% within two days in its absence^[^^80^^]^. The aim is to prevent aortic rupture and relieve regurgitation - this can be done by excision of the intimal tear or by reconstitution of the aorta with a graft^[^^75^^]^. Thirty-day/in-hospital mortality for type A acute AD was demonstrated to be 13% to 17% (median, 14%) for open surgical procedure and 0%-16% (median, 7%) for TEVAR.

Although IMH has the same modality of categorisation as AD, optimal management strategies have not been established. However, Mussa et al.^[^^77^^]^ recommends open surgery for complicated IMH type A, TEVAR for complicated IMH type B, and medical treatment for uncomplicated type A and B, with all types requiring close monitoring to prevent disease progression to AD^[^^81^^]^. The comparison of 30-day/in-hospital mortality of IMH type A and B patients between therapies was the following: medical management (4%-19%; median, 8%), open surgical repair (11%-24%; median, 17%), TEVAR (0%-6%; median, 2%)^[^^77^^]^.

There have been but a few studies which have researched the optimal management of PAU and according to current evidence, a conservative approach is recommended, with open surgery reserved for patients with escalation in pain, increase in ulcer or aortic size, and rupture. For rare type A PAU, urgent surgical intervention is recommended^[^^82^^]^. Type B PAUs represent over 90% of cases and are well localised, therefore they are suited for endovascular graft treatment to avoid dissection or rupture^[^^81^^]^. Technical success of TEVAR was found to be 98.3% with 4.8% overall 30-day mortality^[^^83^^]^. In the event of rupture, it is important to maintain the haemodynamic condition of patients but despite transfusion and catecholamine, most patients sadly die^[^^75^^]^. Normally, in the event of a rupture, a median sternotomy is performed. If the rupture was in the arch or its distal segment, total arch replacement is performed. If it is in the descending aorta, a left posterolateral thoracotomy with cardiopulmonary bypass or a TEVAR is performed^[^^75^^]^. In all cases, PAU must be monitored for disease progression to IMH and AD^[^^82^^]^.

In terms of vasculitis, the cause of aortitis dictates management. This management aims to treat the underlying inflammation, causation, and any arterial complications. For TA and GCA, oral glucocorticoid therapy is a gold standard. Prednisone is first line, with a starting dose of 40-60 milligrams daily for GCA and 1 milligram/kilogram of body weight daily for TA^[^^24^^]^. This dose is gradually adjusted over time whilst symptoms are closely monitored along with inflammatory biomarkers, vascular signs, and radiological signs. This corticosteroid treatment usually lasts months or years in order to achieve complete success^[^^24^^]^. If there is aneurysmal involvement in either disease, it should be monitored for expansion and if required, surgical correction such as open aortic reconstruction may be performed; the indications of which are similar to other aforementioned conditions. Furthermore, surgery may be more beneficial when utilised in specific stages of the progression of these vasculitides. In TA, surgery increases the long-term survival of patients with stage three TA (major complication and progressive disease, based on prognostic classification by Ishikawa and Maetani)^[^^84^^]^. However, surgical intervention in stage 1 patients (no major complications and no evidence of progressive disease), decreased survival due to surgery-related complications^[^^84^^]^. Therefore, medical management is recommended for patients with stage 1 and 2 diseases^[^^84^^]^. In terms of GCA, revascularisation procedures (bypass surgery, angioplasty, or stenting) due to arterial stenosis are required rarely. When these procedures have been performed, restenosis was commonly observed^[^^85^^]^. Finally, the revascularisation procedures should be performed in the quiescent phase of the vasculitis rather than the active, and in experienced centres for optimal outcomes.

## CONCLUSION

Understanding arterial pathologies can have a significant impact on their investigation, diagnosis, and management. Screening for such pathologies will help predict adverse and life-threatening events, which could be treated prophylactically.

**Table:** 

Authors' roles & responsibilities	
AH	Substantial contributions to the conception or design of the work; or the acquisition, analysis, or interpretation of data for the work; drafting the work or revising it critically for important intellectual content; final approval of the version to be published
PAS	Substantial contributions to the conception or design of the work; or the acquisition, analysis, or interpretation of data for the work; drafting the work or revising it critically for important intellectual content; final approval of the version to be published
KH	Substantial contributions to the conception or design of the work; or the acquisition, analysis, or interpretation of data for the work; drafting the work or revising it critically for important intellectual content; final approval of the version to be published
AP	Substantial contributions to the conception or design of the work; or the acquisition, analysis, or interpretation of data for the work; drafting the work or revising it critically for important intellectual content; final approval of the version to be published
